# Air-Coupled Reception of a Slow Ultrasonic A_0_ Mode Wave Propagating in Thin Plastic Film

**DOI:** 10.3390/s20020516

**Published:** 2020-01-16

**Authors:** Rymantas J. Kazys, Almantas Vilpisauskas

**Affiliations:** Ultrasound Research Institute, Kaunas University of Technology, K. Baršausko St. 59, LT-51423 Kaunas, Lithuania; almvilp@gmail.com

**Keywords:** air-coupled ultrasound, contactless ultrasonic sensors, guided ultrasonic wave, subsonic A_0_ Lamb wave, air-coupled ultrasonic array, evanescent wave, air-coupled reception of subsonic guided wave

## Abstract

At low frequencies, in thin plates the phase velocity of the guided A_0_ mode can become slower than that of the ultrasound velocity in air. Such waves do not excite leaky waves in the surrounding air, and therefore, it is impossible to excite and receive them by conventional air-coupled methods. The objective of this research was the development of an air-coupled technique for the reception of slow A_0_ mode in thin plastic films. This study demonstrates the feasibility of picking up a subsonic A_0_ mode in plastic films by air-coupled ultrasonic arrays. The air-coupled reception was based on an evanescent wave in air accompanying the propagating A_0_ mode in a film. The efficiency of the reception was enhanced by using a virtual array which was arranged from the data collected by a single air-coupled receiver. The signals measured at the points corresponding to the positions of the phase-matched array were recorded and processed. The transmitting array excited not only the A_0_ mode in the film, but also a direct wave in air. This wave propagated at ultrasound velocity in air and was faster than the evanescent wave. For efficient reception of the A_0_ mode, the additional signal-processing procedure based on the application of the 2D Fourier transform in a spatial–temporal domain. The obtained results can be useful for the development of novel air-coupled ultrasonic non-destructive testing techniques.

## 1. Introduction

Ultrasonic guided Lamb waves are widely used for the non-destructive testing and evaluation of various objects and structures such as composite plates [1,2], metal pipes [3], composite panels [4], and paper products [5,6,7].

Usually, guided waves are excited by an ultrasonic transducer coupled to a test structure by a coupling liquid. However, there are cases when such an approach is not suitable because the tested material may be damaged or contaminated by the coupling liquid. In addition, contact type transducers are not suitable for measurements of moving structures, for example during a manufacturing process. Typical examples of such materials are paper, wood, and some plastic and aerospace components. In these cases, air-coupled excitation and the reception of Lamb waves are used.

One of the contactless methods used for excitation and registration of guided waves is application of lasers [8,9,10,11,12,13]. Excitation of ultrasonic waves in solids in this case is due to thermal expansion of the solid surface caused by heating with the laser optical beam or due to the ablation phenomenon. The second approach may significantly damage the surface of the object, therefore usually for measurement purposes the thermal expansion is used. Laser ultrasonics is already exploited for inspection of composite structures used in aerospace [13]. As it was noted in [14] generation of ultrasonic waves by lasers is a rather low-efficiency technique.

For detection of ultrasonic vibrations interferometric techniques are usually used. The drawback of these techniques is that they are not suitable for rough surfaces. Therefore, a type of non-destructive testing was proposed that combined laser excitation with air-coupled reception of ultrasonic waves by piezoelectric or electrostatic transducers [15,16]. The main shortcoming of laser techniques is that they are rather expensive.

Air-coupled excitation, propagation, and reception of guided waves were discussed in detail in [14,17,18,19]. Usually, for such purposes, a pitch-catch configuration of air-coupled ultrasonic transducers is used. The problems associated with this case, such as reflections in the air-gap and misalignment of air-coupled probes, were analyzed in [20,21]. The air-coupled ultrasonic technique has been used for defect detection in composite materials [14,18,19,22], polymer pipes [23], and cardboard tubes [24] and for the non-destructive testing of square-shaped CFRP composite rods [25]. They enable not only detection of delamination type defects, pores and other non-uniformities, but also measurement of their position and dimensions. Usually these techniques are based on exploitation of mode-conversion phenomena.

In most cases for the non-destructive testing of plate-type structures, the lowest antisymmetric Lamb wave mode A_0_ is exploited because its amplitude is the highest among the other modes, and it is sensitive to various types of defects [26]. The testing method is commonly based on the assumption that the guided wave in the test structure is faster than the ultrasonic wave in air [27]. In this case, the A_0_ mode is excited and picked up by ultrasonic transducers deflected with respect to the plate according to Snell’s law [6]. In the case of an air-coupled multi-element array, the necessary deflection angle of the ultrasonic beam can be adjusted electronically by applying properly delayed excitation signals to the array elements [28]. However, the phase velocity *c*_A0_ of the A_0_ mode is frequency dependent. For free isotropic homogeneous plates and low frequencies, the phase velocity is given by [6]:

(1) $$c_{A0}^{2}=\sqrt{\frac{E}{12\rho \left(1-\nu^{2}\right)}}2\pi fd$$

where *E* is the Young’s modulus, *ρ* is the density, *ν* is the Poisson’s ratio, *f* is the frequency, and *d* is the thickness of the plate. From Equation (1), it follows that the phase velocity *c*_A0_ depends on the *fd* values. At *fd* values *≥*500 and materials with high Young’s modulus values, such as aluminum foil and composite materials, the phase velocity is higher than the ultrasound velocity in air, and the Lamb waves in thin films are excited according to Snell’s law [29]. At lower *fd* values at low frequencies, in the thin plates for some materials, *f* and/or the A_0_ mode phase velocity can become slower than the ultrasound velocity in air. In this case, the materials should possess a low ratio of Young’s modulus to density *E*/*ρ*. This requirement is fulfilled in polymer- and glass-type materials, and some composite materials [30]. The Poisson’s ratio *ν* is insignificant in this case because it is usually smaller than 0.5 [31].

**Figure 1:**
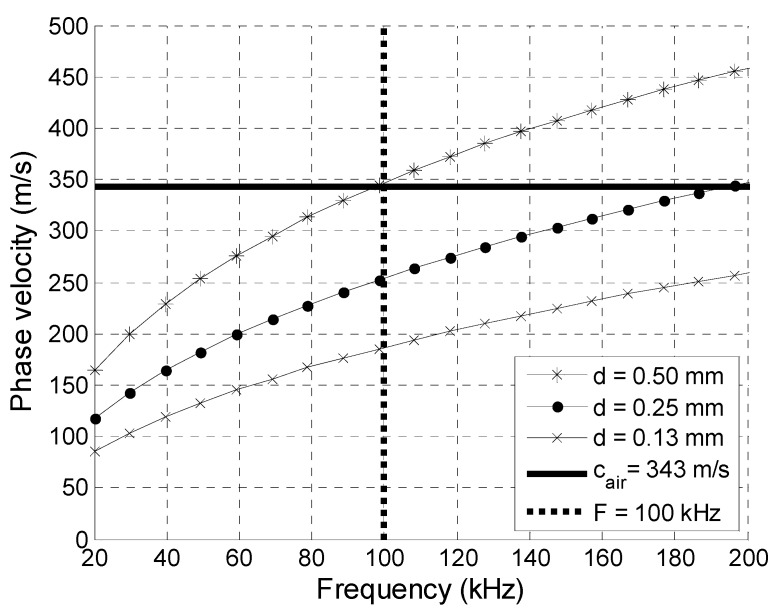
Phase velocities of A_0_ Lamb wave mode in clear PVC films of different thicknesses *d.*

Clear polyvinyl chloride (PVC) films used for packaging and Corning^®^ Gorilla^®^ Glass plates (Corning Incorporated, Corning, NY, USA) used to protect smartphone screens are examples of such materials. We calculated the A_0_ Lamb wave mode phase velocity in PVC films and Corning^®^ Gorilla^®^ Glass 6 plates of different thicknesses by a semi-analytical finite element method applied on isotropic single-layer plates, air-loaded on both sides [32]. The plate cross-section profile of a chosen different thickness d was divided into six layered finite elements. The other material parameters used for calculations are presented in Table 1 [33,34]. The modelling results for clear PVC films are shown in Figure 1. The ultrasound velocity in air, c_air_ = 343 m/s, is indicated by a solid horizontal line, and the vertical dashed line at a frequency of 100 kHz limits the zone where the A_0_ mode phase velocity is slower than the velocity in air.

**Figure 2:**
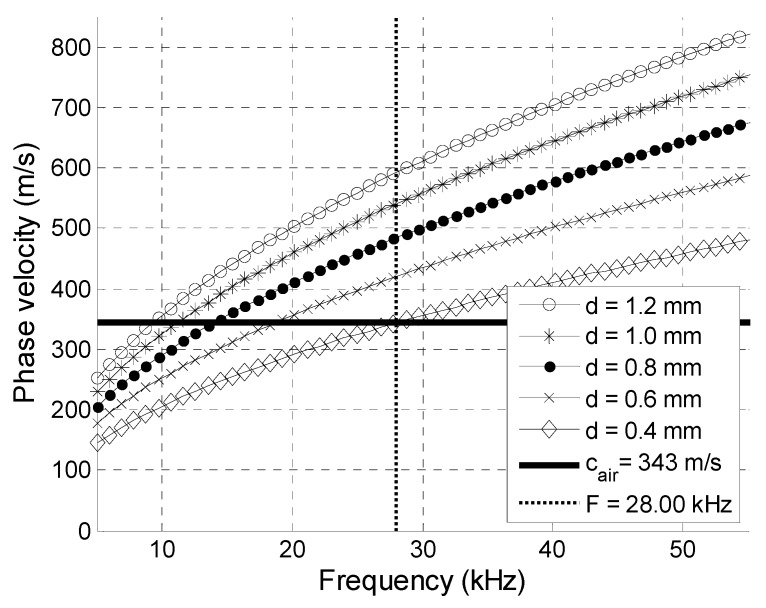
Phase velocities of A_0_ Lamb wave mode in Gorilla^®^ Glass 6 plates of different thicknesses *d*.

The Corning^®^ Gorilla^®^ Glass 6 possesses a much higher Young’s modulus than that of the PVC material (Table 1), but, in spite of that, there was a frequency range up to 28 kHz in which the A_0_ mode phase velocity was slower than the velocity in air (Figure 2).

The obtained data show that there are various plate-type materials in which the velocity of A_0_ mode is slower than that in air. Such waves are called subsonic or slow Lamb waves. In the slow A_0_ Lamb wave mode case, there is no leaky ultrasonic wave, which means that it is not possible to excite and receive such a wave by ultrasonic transducers deflected at an angle according to Snell’s law. To overcome this limitation, we used linear air-coupled ultrasonic arrays to excite the slow A_0_ Lamb wave mode. This method has been analyzed in detail in our previous papers [35,36]. However, the air-coupled reception of such waves encounters significant problems because the lack of ultrasonic wave leakage into the surrounding air has not yet been solved.

To solve this problem, we accompanied the guided A_0_ mode with an evanescent wave that propagated in air close to the surface of the plate or film in which the A_0_ mode was propagated. To our knowledge, evanescent waves have not yet been applied for the air-coupled reception of guided waves, and this is the main topic of this paper. However, such use of an evanescent wave is not straightforward because it is usually weaker that the wave propagating directly in air from a source of ultrasonic waves. The velocity of this interfering wave is equal to the ultrasound velocity in air and is much faster than the movement velocity of the evanescent wave, which is equal to the velocity of the slow A_0_ mode. Therefore, it is necessary to pick up the slower evanescent wave that is masked by the faster and stronger direct wave. To solve this problem, we applied special air-coupled ultrasonic arrays and signal-processing procedures to separate a relatively weak evanescent wave signal from stronger ultrasonic waves propagating directly through air.

Attenuation of ultrasonic waves in plastic materials with an increasing frequency is increasing and at 500 kHz can reach 5 dB/cm [37]. At lower frequencies at which the sub-sonic A_0_ mode may propagate this attenuation is lower and in the case of PVC at 44 kHz is 2 dB/cm. [38]. Hence, one advantage of the sub-sonic A_0_ mode is the lower attenuation.

During the manufacturing of PVC films, various defects such as wrinkles, holes, a rough surface and thickness variations arise. Some of these are detected by optical methods, but such defects as holes and, especially, thickness variations, could be found by ultrasonic methods using the discussed A_0_ mode guided waves [38,39]. Taking into account that the velocity of the A_0_ mode can be measured with a high precision such a method can be used for measurements of thickness of moving plastic ribbons.

The objective of this research was the development of a novel ultrasonic air-coupled technique for the reception of the slow A_0_ Lamb wave mode in a thin plastic film based on the pick-up of evanescent waves by means of a special multi-element ultrasonic array and signal-processing procedure.

The paper is organized as follows. In Section 2, the theoretical modelling of the slow A_0_ mode in a thin PVC film is presented by highlighting the 2D spatial distributions of normal displacements in the reception area. In Section 3, the theoretical results are verified by experimental investigations using a laser interferometer. In Section 4, the results of the evanescent wave field modelling are presented, and the requirements for the air-coupled receiving transducer are formulated. In Section 5, the reception of the slow A_0_ mode by means of the proposed virtual linear array is described. For the separation of the A_0_ mode signal from a strong direct wave in air, the proposed signal-processing procedure is presented. Experimental results confirming the efficiency of the proposed reception technique are described.

## 2. Theoretical Analysis

For the air-coupled reception of the slow A_0_ mode propagating in thin films, we used a linear multi-element array. To evaluate the efficiency of this approach, theoretical modelling was performed to obtain 2D spatial distributions of the normal displacements in the area corresponding to the geometry of the receiving air-coupled array. In our previous work devoted to the excitation but not the reception of the slow A_0_ mode, only displacements along one line corresponding to the symmetry axis of the transmitting array were calculated [35]. However, real arrays possess finite dimensions because the spatial distributions in the plane of the analyzed film are not uniform, and the achievable efficiency of the air-coupled array is not clear. Therefore, the main objective of our modelling was to determine the 2D distributions of the normal displacements in the reception area and to evaluate the performance of the receiving array. For our investigations, we selected a thin PVC film with a thickness of 130 μm.

**Figure 3:**
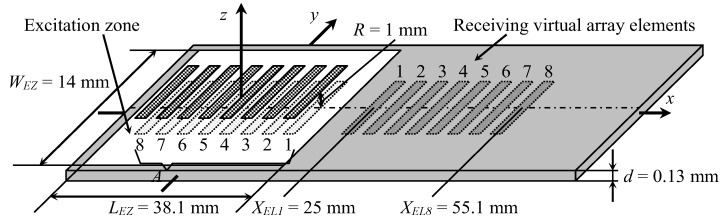
Simulation of reception of slow A_0_ Lamb wave mode by air-coupled multi-element array.

For the excitation, a planar phased array consisting of eight elements was used (Figure 3). The dimensions of the radiating aperture of a single array element were 7 × 1 mm. The coordinates of the centers of the exciting array elements were the same as those in [35]. The distance between the radiating air-coupled array and the PVC film was 1 mm. Each element radiated at 22.62 kHz with a 40-period duration signal with the Gaussian envelope fronts.

The air-coupled excitation of the slow A_0_ Lamb wave mode in a PVC film by a multi-element array was simulated using the impulse response method [40,41,42], and the propagation of the excited guided wave in the film was simulated by the time harmonic solution method. These methods were realized in a free software tool “The Lamb Matlab toolbox” (Beta version 0.1) [43]. The impulse response method allowed the calculation of the radiation of ultrasonic waves by rectangular elements of the air-coupled array through the air gap between the array and the PVC film. As a result, the 2D spatial distributions of the acoustic pressure on the surface of the PVC film were obtained. The pressure distributions were used to model the excitation and propagation of the guided wave in a PVC film.

Reception was based on the exploitation of the evanescent wave in air accompanying the propagating guided wave in close vicinity to the PVC film surface. The evanescent wave in the surrounding air was created by normal film displacements; therefore, knowledge of the spatial distributions of the normal displacements of the film surface was required. We have shown [35,36] that air also propagates a direct wave radiated by the transmitting array which affects the guided wave propagating in the film. As a result, these two waves interfere with each other in the PVC film and cause periodic oscillations of the amplitude of normal displacements in space. However, by using the time harmonic solution modelling method, we can investigate the reception process, without the influence of the undesired airwaves, to estimate the potential performance of a receiving array. A virtual eight-element receiving array was created on the PVC film surface at a certain distance from the excitation array (Figure 3). Eight rectangular zones on the surface of the PVC film were selected with dimensions of 7 × 1 mm, which were equal to dimensions of the apertures of the receiving elements in the array. The coordinate of the first receiving array element center was *X_EL_*_1_ = 25 mm. The distance between array element centers was chosen according to the Lamb wavelength as 4.3 mm; therefore, the spacing between elements was 3.3 mm. In these zones, the normal displacement signals were calculated and used to form the array signal according to the selected signal-processing algorithm.

**Figure 4:**
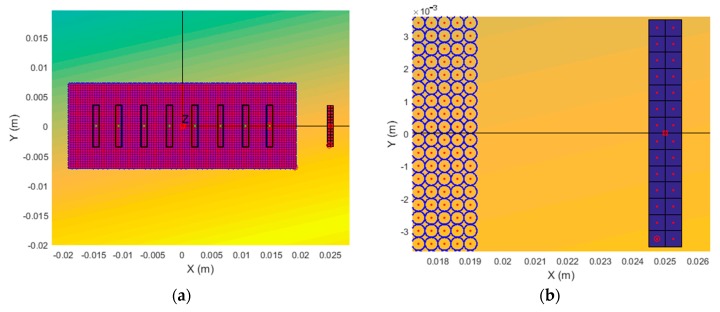
Image of excitation area and first receiving virtual element: (**a**) general view and (**b**) zoomed image of receiving zone in vicinity of receiving element 1.

During simulations, the excitation zone shown in Figure 3 by the white rectangle was divided into 3456 circular subzones with diameters of 0.4 mm (Figure 4a). The simulations were performed in the frequency range 10–40 kHz. To obtain an efficient excitation of the slow A_0_ mode, the delay times of the excitation signals of the transmitting array were optimized according to our proposed algorithm [35].

Using this approach, the air pressure signals acted on the film surface only in the excitation zone, and the virtual receiving array elements were protected from unwanted airwaves. The modelling results were verified by measuring the normal displacements with a laser interferometer. The results are presented in Section 3.

Each virtual 7 × 1 mm aperture was divided into 28 quadratic zones, and normal displacement signals were calculated in the centers of those zones. The division step was 0.5 mm, which means that there were two columns of zones across the element and 14 rows along the length of the zone (Figure 4b).

**Figure 5:**
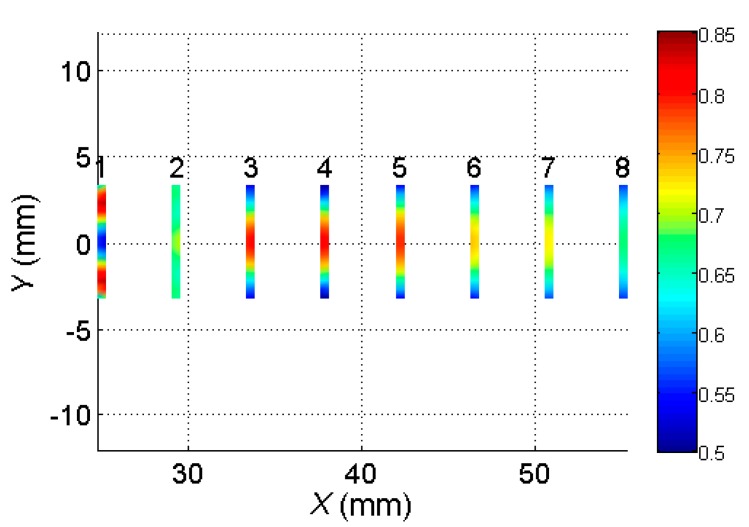
Spatial distribution of maximum normal displacement amplitudes of virtual receiving elements 1–8. The color bar shows the amplitudes (µm) of the normal displacements.

The simulated spatial distributions of maximum normal displacement amplitudes of the virtual receiving elements 1–8 are shown in Figure 5.

It follows from the modelling results that the 2D spatial distributions of the normal displacements in the zones of the virtual receiving elements were non-uniform. The waveforms calculated at all points in the areas covered by the virtual elements formed the received signals at the outputs of those elements. To obtain those signals in the zone of each virtual array element, the simulated waveforms were averaged as

(2) $$U_{averaged}\left(t\right)=\frac{\overset{N_{S}}{\underset{i=1}{\sum }}U_{i}\left(t\right)}{N_{S}}$$

where *N_S_* is the number of calculated waveforms in the area of one virtual array element, and *U_i_*(*t*) is the simulated waveform of the normal displacement at a particular point *i* of the virtual element.

**Figure 6:**
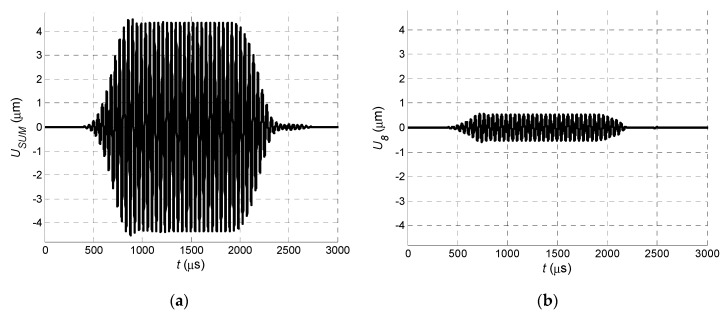
Waveforms of normal displacements: (**a**) at the output of the entire receiving array and (**b**) at the output of receiving array Element 8.

The signal at the output of the entire virtual array was obtained by adding the signals at the outputs of all virtual elements with proper delays. The ultrasonic signal at the output of the receiving array was obtained by adding the delayed signals at the outputs of the individual array elements as

(3) $$u_{out}\left(t\right)=\overset{N}{\underset{j=1}{\sum }}u_{averaged}^{j}\left(t-j\Delta t\right)$$

where *j* is the number of a particular array element, *N* is the total number of array elements, and Δ*t* is the introduced delay of the received signal between the neighboring elements. In the analyzed case, the signal delay between the neighboring elements was selected as Δ*t* = 50.4 μs, which corresponds to the A_0_ mode propagation time between the elements in the PVC film. The signal obtained at the output of the virtual receiving array is shown in Figure 6a. As a comparison, the signal at the output of array Element 8 is shown in Figure 6b.

**Figure 7:**
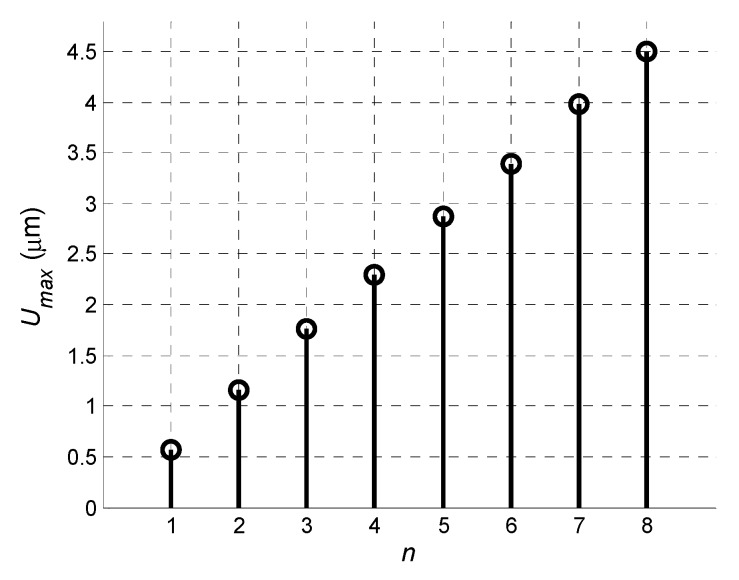
Amplitude of received signal *U_max_* at output of receiving array versus number of array elements *n*.

The dependence of the signal amplitude at the output of the virtual array versus the number of array elements is shown in Figure 7. Despite the non-uniform distributions of the normal displacements, the application of the virtual array provided a significant improvement in the reception process.

The presented model did not take into account the direct ultrasonic wave radiated in air by the excitation array, which affects the reception of a guided wave by an air-coupled receiver. However, the obtained results show the achievable possibilities of the application of an air-coupled receiving array.

## 3. Experimental Investigation by Laser Interferometer

**Figure 8:**
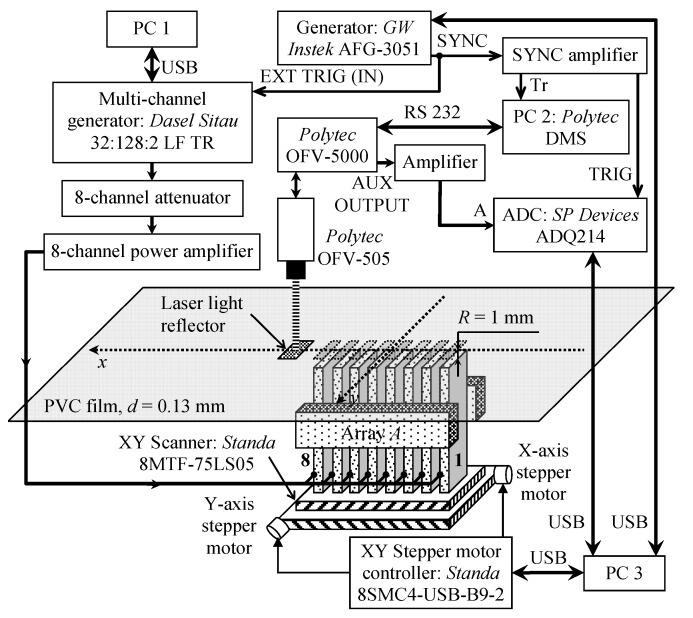
Experimental setup using a laser interferometer.

**Figure 9:**
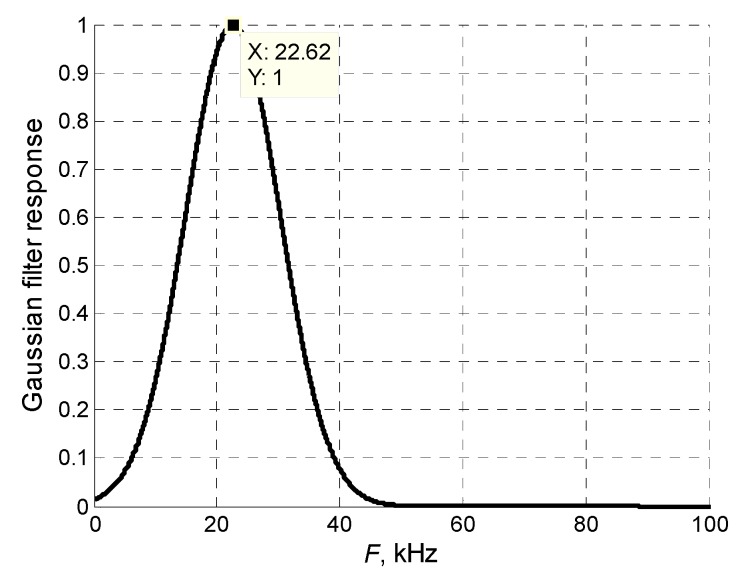
Frequency response of the Gaussian filter.

The numerical simulation results were verified by experimental investigations performed with a laser interferometer. The experimental setup is shown in Figure 8. The ultrasonic guided wave was excited in the PVC film by the air-coupled eight-element array via a 1 mm air gap. The array was excited by 40 negative square-wave pulses with 23 V amplitude. The electric excitation signals were formed by three electronic units: a multi-channel generator Dasel Sitau 32:128:2 LF TR (DASEL Systems, Madrid, Spain), an eight-channel attenuator, and an eight-channel amplifier (both are from Ultrasound Research Institute, Kaunas, Lithuania). The 3.6 kΩ output impedance of the generator was too high for efficient array excitation, and an additional electronic unit was used to reduce the output impedance. The eight-channel attenuator was used to reduce and adjust the signal amplitude. After the attenuator, the pulses were transferred to the eight-channel amplifier with a low 5 Ω output impedance. For the efficient excitation of the slow A_0_ mode, the experimentally determined [35] delay times between array elements were created by the generator. The normal displacements of the PVC film were measured by a Polytec OFV-5000 laser interferometer (Polytec GmbH, Waldbronn, Germany). After conversion to a digital format by the analogue-to-digital converter ADQ 214 (Teledyne Signal Processing Devices Sweden AB, Linköping, Sweden), the signals were recorded and stored in a PC 3 personal computer. The ultrasonic array was fastened to a scanner Standa 8MTF-75LS05 (Standa Ltd., Vilnius, Lithuania) and moved along the *x*-axis below the PVC film to obtain a B-Scan of the normal displacements. The scanner performed the B-scan along the *x*-axis in the range of 50 mm in 0.5 mm steps. To reduce the influence of noise at each point, 50 measurements were performed and averaged. The averaged signals were filtered by a Gaussian filter, the frequency response of which is shown in Figure 9.

**Figure 10:**
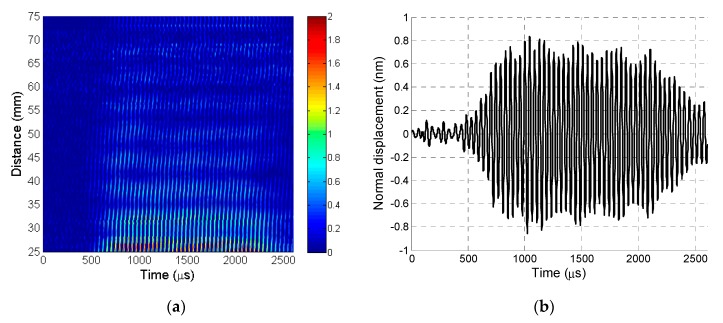
(**a**) B-scan recorded by laser interferometer before 2D filtering. (**b**) A-scan signal taken from B-scan before 2D filtering at a distance of 37.5 mm.

The recorded B-scan is presented in Figure 10a. The A-scan signal was taken from the B-scan at a distance of 12.5 mm, as shown in Figure 10b.

To determine the type of wave received, its phase velocity was determined from the recorded B-scan. This was performed by choosing two different points, *P*_1_(*x*_1_, *t*_1_) and *P*_2_(*x*_2_, *t*_2_), in the same B-scan line, where *x*_1_ and *x*_2_ correspond to the measurement distances, and *t*_1_ and *t*_2_ correspond to the time instants at those spatial points. The phase velocity was found as

(4) $$v=\frac{\Delta x}{\Delta t}=\frac{x_{2}-x_{1}}{t_{2}-t_{1}}$$

The calculated phase velocity of 338.7 ± 0.5 m/s was much higher than the expected velocity of the slow A_0_ mode and slightly lower than the sound velocity in air (*v_air_* = 343 m/s). Therefore, the resulting normal displacements recorded by the laser interferometer were caused by the interference of the slow A_0_ Lamb wave mode and the film vibrations caused by a strong direct airwave. A similar experiment found that acoustic shielding at such low frequencies is not efficient and does not significantly reduce the direct ultrasonic wave propagating in air from an air-coupled array [36].

Therefore, for a more efficient separation of the slow A_0_ Lamb wave mode and the evanescent wave, we used the 2D spatial–temporal filtering of the recorded B-Scan. This method was based on the use of different wave propagation velocities.

In this case, the 2D Fourier transform *FFT*_2*D*_ was applied to the recorded B-scan data *u*(*t*, *x*) as

(5) $$U\left(f,\lambda^{-1}\right)=FFT_{2D}\left[u\left(t,x\right)\right]$$

**Figure 11:**
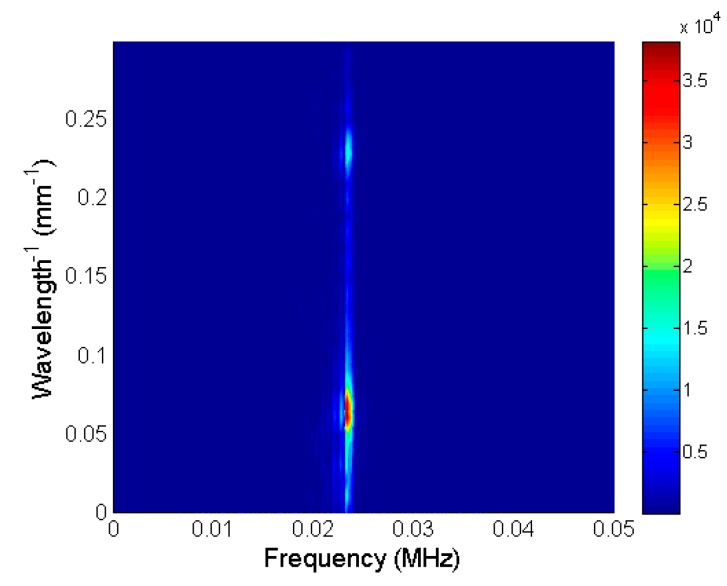
2D spatial–temporal spectrum of recorded B-scan before filtering.

The obtained 2D spatial-temporal spectrum is shown in Figure 11. The highest amplitudes in this spectrum were concentrated at a frequency of 22 kHz and spread along the *λ*^−1^ spatial frequencies. The lowest spatial frequencies in the range 0.05–0.1 mm^−1^ corresponded to the strong direct wave propagating in air. The weaker evanescent wave was in the range 0.2–0.25 mm^−1^. This wave can be separated by a 2D spatial–temporal band-pass filter with the spatial–temporal response *H_f_* (*f*, *λ*^−1^) as:

(6) $$U_{f}\left(f,\lambda^{-1}\right)=U\left(f,\lambda^{-1}\right)\cdot H_{f}\left(f,\lambda^{-1}\right)$$

**Figure 12:**
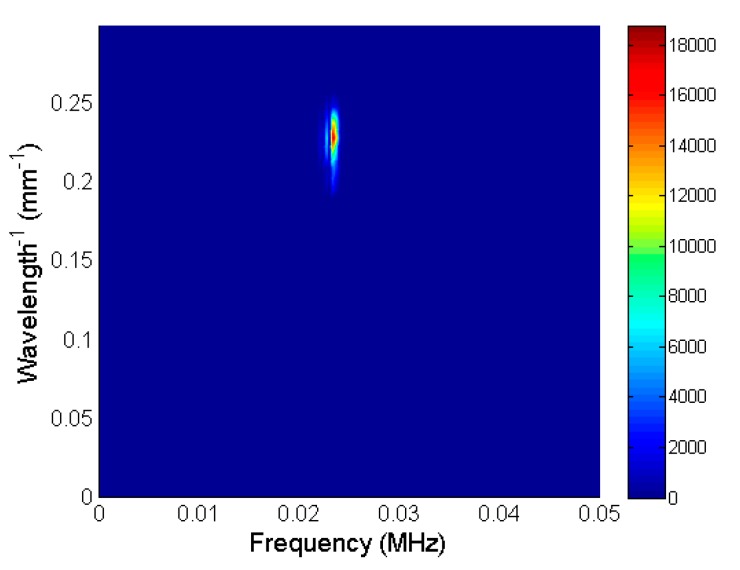
2D spatial–temporal spectrum of recorded B-scan after filtering.

For the separation of the evanescent wave, the proper cut-off frequencies of the 2D band-pass filter were selected. The best results were obtained when the frequency range 22–24.6 kHz was chosen and the spatial frequency range *λ*^−1^ was 0.2–0.25 mm^−1^ (Figure 12). The filtered B-scan data in the time–distance domain *u_f_*(*t*, *x*) were obtained by performing the inverse 2D Fourier transform as:

(7) $$u_{f}\left(t,x\right)=FFT_{2D}^{-1}\left\lfloor U_{f}\left(f,\lambda^{-1}\right)\right\rfloor$$

**Figure 13:**
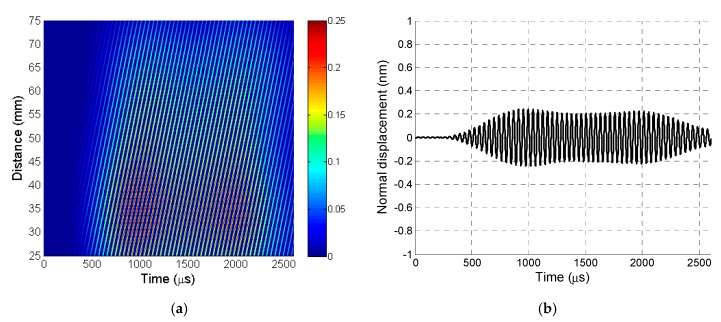
(**a**) B-scan recorded by laser interferometer after 2D filtering. (**b**) A-scan signal taken from B-scan after 2D filtering at a distance of 37.5 mm.

The filtered B-scan is shown in Figure 13a. The A-scan signal taken from the filtered B-scan at a distance of 37.5 mm is shown in Figure 13b.

Comparison of the signals shown in Figure 10b and Figure 13b shows that the A_0_ mode signal amplitude obtained after 2D filtering was about four times lower than the displacements caused by the slow A_0_ Lamb wave mode and the film vibrations caused by a strong direct air wave. The interferometric measurements revealed the influence of the air wave on the vibrations of the film because only the normal displacements of the PVC film surface were measured and not the direct wave in air.

In addition, the obtained waveforms indicate that the A_0_ mode signal and the signal caused by the direct air wave were completely overlapping, and the proposed signal procedure must be applied for their separation.

Measurement of the phase velocity according to the above-described method provided a velocity of 102.2 ± 0.5 m/s, which corresponds to the propagation velocity of the A_0_ mode in PVC film. The obtained result confirms the efficiency of the proposed wave separation method based on 2D spatial–temporal filtering. This signal-processing method was also used for the separation of an evanescent wave accompanying the A_0_ mode in a strong direct wave in air.

## 4. Application of Evanescent Waves

**Figure 14:**
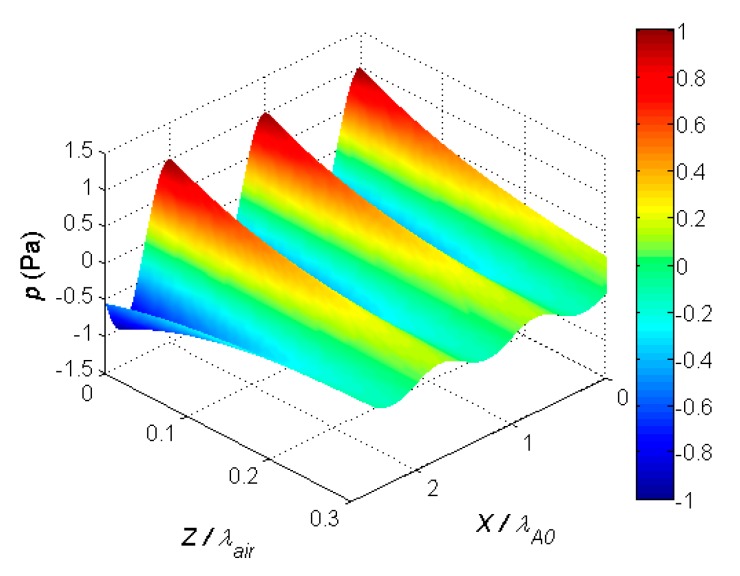
3D spatial structure of evanescent wave field in air above PVC film.

The slow A_0_ Lamb wave mode propagating in the PVC film creates an accompanying evanescent wave in the surrounding air. This wave can be used for contactless reception of the A_0_ mode. Let us analyze the conditions that should be fulfilled in this case. An evanescent wave is created by normal displacements of the film caused by the A_0_ mode and moves in the air along the film surface with the velocity of the A_0_ mode. The acoustic pressure of the evanescent wave decays exponentially with the distance to the film surface [44] as

(8) $$p\left(x,z,t\right)=Ae^{-k_{z}z}e^{-i\left(\omega t-k_{x}x\right)}$$

where *p*(*x*, *z*, *t*) is the acoustic pressure field in air, *x* is the coordinate on the film surface along which the slow A_0_ Lamb wave mode travels, *k_x_* = 2*π*/*λ*_A0_ is the wave number in the plate along the Lamb wave propagation direction, *z* is the coordinate in air perpendicular to the film surface, *k_z_* = 2*π*/*λ_air_* is the wave number in air, *t* is time, *A* is the pressure amplitude, and *ω* is the angular frequency. Let us assume that the pressure amplitude *A* at the film surface is 1 Pa. The wavelength in the film *λ*_A0_ at the frequency 22.62 kHz is 3.8 mm, and the wavelength in air *λ_air_* is 15.2 mm. The calculated 3D spatial structure of the evanescent wave field is shown in Figure 14. The distances in air *z* and on the film surface *x* were normalized by the corresponding wavelengths *λ*_A0_ and *λ_air_*.

The results show that, along the x-direction, the variations of the acoustic pressure were periodic, with the spatial period equal to the wavelength of the slow A_0_ Lamb wave mode propagating in the PVC film. Along the z-direction perpendicular to the film surface, the evanescent wave in air decayed very quickly; at the distance *z*/*λ_air_* = 0.2, the pressure amplitude was almost 10 times lower. In absolute but not normalized units, the decay increased with an increase in the frequency of the guided wave.

Considering these results, it is possible to formulate the requirements for the contactless reception of the slow-guided A_0_ mode. To avoid the integration of evanescent wave signals with opposite phases, the dimension of the receiving aperture along the x-direction should be at least smaller than the quarter wavelength (<*λ*_A0_/4) of the slow A_0_ Lamb wave mode in the film. The distance in air between the receiving transducer and the film surface should be as short as possible (<0.1 *λ_air_*). There are no specific requirements for the dimensions of the receiving aperture across the x-direction.

## 5. Experimental Investigation of Air-Coupled Reception of Slow A_0_ Mode

**Figure 15:**
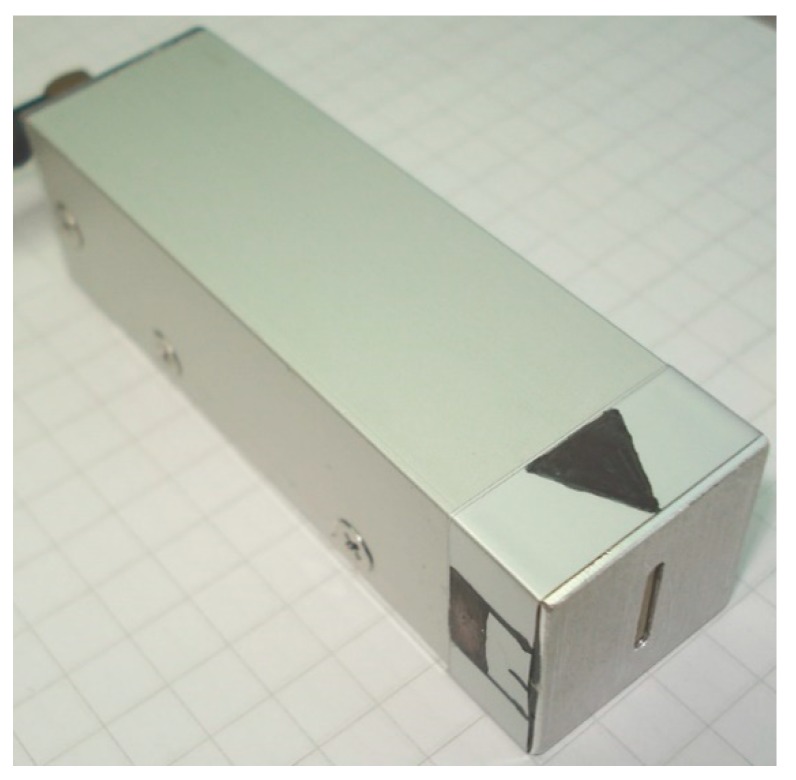
Air-coupled transducer for non-contact reception of evanescent waves.

**Figure 16:**
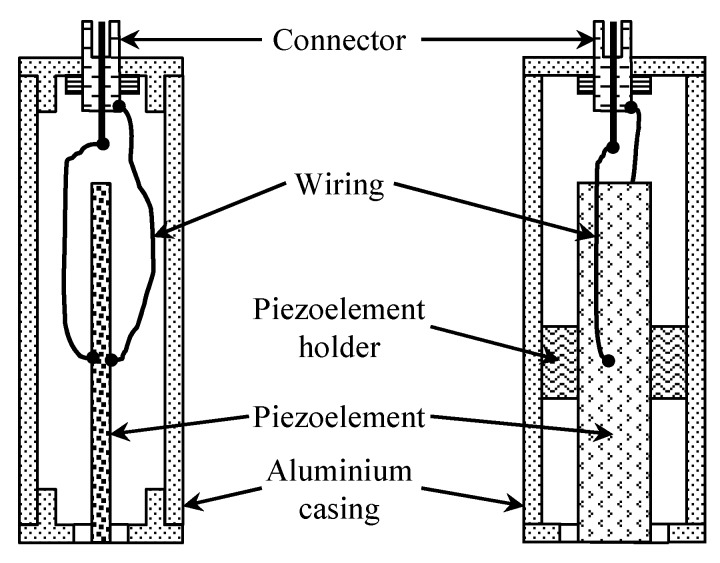
Cross-sections of the manufactured air-coupled transducer in two orthogonal planes.

To check the possibility of the air-coupled reception of the slow A_0_ Lamb wave mode by using an evanescent wave in air, another experimental investigation was performed. For the non-contact reception of ultrasonic waves, the air-coupled transducer designed according to the conclusions of the presented analysis of evanescent waves is shown in Figure 15. The design of the transducer is presented in Figure 16.

The active element of the transducer was a 60 × 11 × 1 mm piezoelectric strip made of piezoelectric ceramic Pz29 vibrating in a transverse extensional mode. For the reception of the ultrasonic evanescent wave, the dimensions of the aperture tip of the strip were 1 × 11 mm to satisfy the requirements of the theoretical analysis, which state that the width of the aperture along the slow wave propagation direction should be much less than the wavelength of the slow A_0_ mode. In our case, to fulfil this condition, the width of the aperture was 1 mm and the A_0_ mode wavelength was 4.3 mm. To minimize the influence of electromagnetic noise, the piezoelectric strip was placed in a 1.5 mm thick aluminum casing with dimensions of 84 × 25 × 25 mm. The black triangular mark in Figure 15 indicates the position of the receiving aperture. During the experiments, the transducer was oriented with the longer side of the aperture perpendicular to the propagation direction of the A_0_ mode wave.

**Figure 17:**
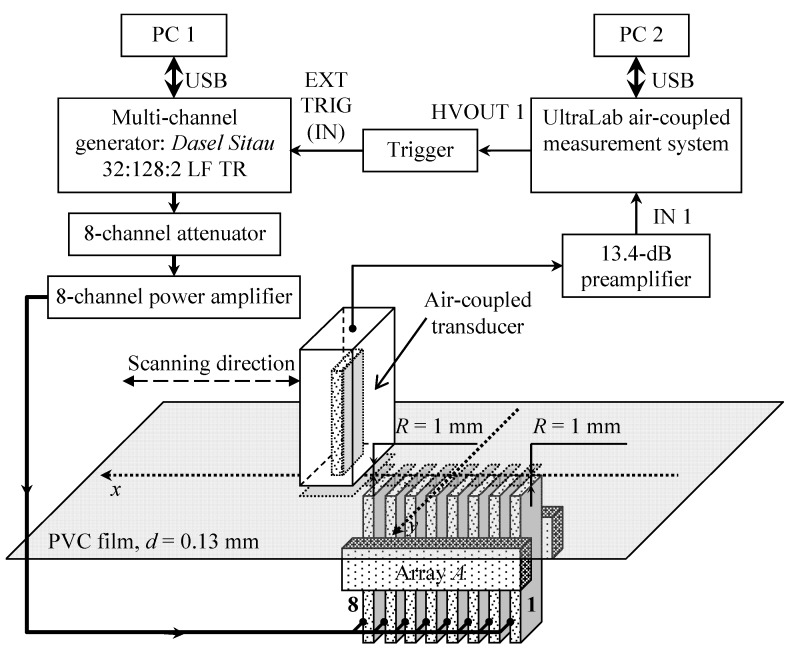
Experimental setup for non-contact generation and reception of A_0_ Lamb wave mode.

The experimental setup used for the air-coupled excitation and reception of the slow A_0_ Lamb wave mode is shown in Figure 17.

**Figure 18:**
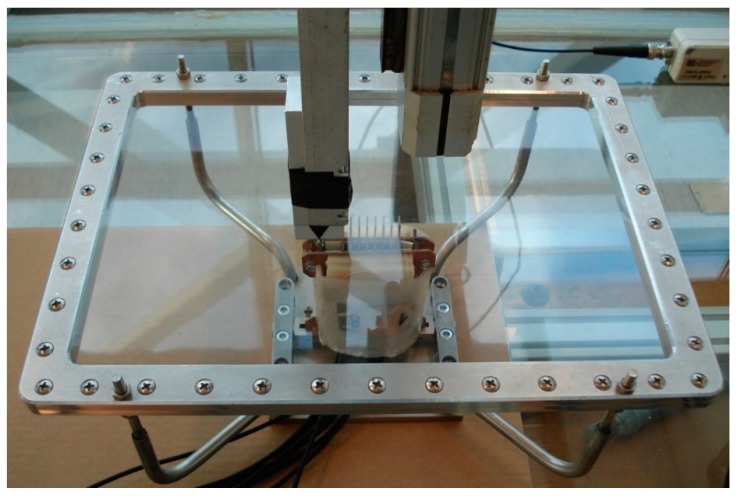
View of PVC film mounting bracket and acoustic part of experimental setup.

The view of the experimental setup part consisting of air-coupled transducers and the PVC film fixed in a mounting bracket is shown in Figure 18.

The air-coupled array and sample of the PVC film were fixed in a special bracket consisting of two parts. The lower part was used to adjust and fix the proper direction of the air-coupled multi-element array used to excite the guided wave in the film. The upper part held the PVC film sample. The eight-element air-coupled acoustic array was excited by 15 negative square-wave pulses with 23 V amplitude. The electric excitation signals were formed using the same three electronic units as in the previous experiment: the multi-channel generator, the attenuator, and the eight-channel amplifier (Figure 17). The single air-coupled transducer described above was used to pick up the evanescent wave caused by the propagating A_0_ mode in the PVC film. The received signals were amplified by a 13.4 dB pre-amplifier and registered by the UltraLab air-coupled measurement system (Ultrasound Research Institute, Kaunas, Lithuania).

**Figure 19:**
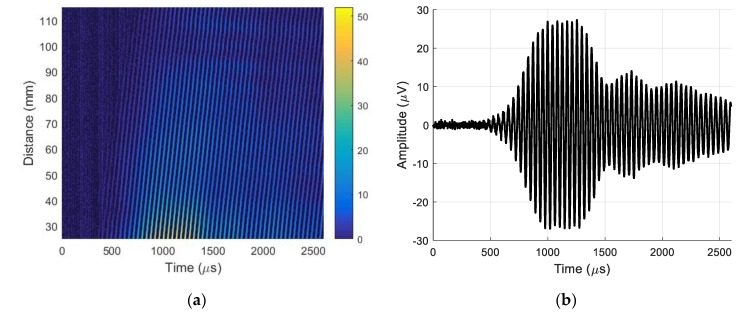
(**a**) B-scan recorded by a single air-coupled ultrasonic transducer before filtering. (**b**) A-scan signal taken from the B-scan before filtering at distance of 37.5 mm.

During the experiments, the air-coupled array was excited by 22.62 kHz pulses with optimized delays between pulses [35]. The receiving transducer was fastened to the scanner of the UltraLab air-coupled system. The scanner performed B-scans along the *x*-axis in the range of 90 mm at 0.1 mm steps. Each temporal signal in the B-scans was obtained by averaging 32 signals. The recorded B-scan is shown in Figure 19a. The A-scan signal taken from the B-scan at a distance of 37.5 mm is shown in Figure 19b.

**Figure 20:**
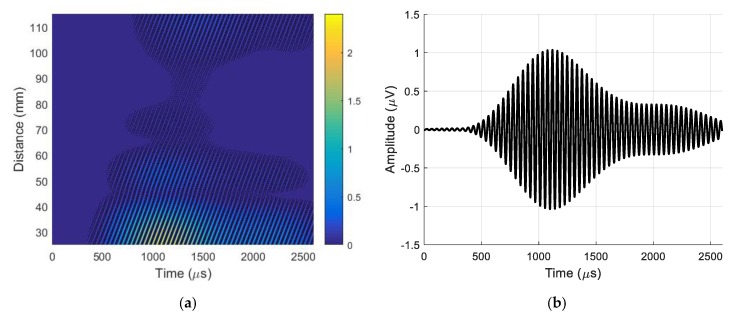
(**a**) B-scan recorded by a single air-coupled ultrasonic transducer after 2D filtering. (**b**) A-scan signal, taken from the B-scan after 2D filtering at distance of 37.5 mm.

To identify the type of wave received, its phase velocity was calculated from the recorded B-scan using Equation (4). The phase velocity of 372.1 ± 0.5 m/s was much higher than the expected velocity of the slow A_0_ mode and slightly higher than the sound velocity in air (343 m/s). This means that the recorded ultrasonic pulses propagating in the air were significantly stronger than the evanescent waves created by the slow A_0_ Lamb wave mode. The evanescent waves were separated by using the 2D spatial–temporal filtering of the recorded B-scan described in Section 3. The filtered B-scan is shown in Figure 20a. The A-scan signal taken from the filtered B-scan at a distance of 37.5 mm is presented in Figure 20b. Comparison of the amplitudes of the A-scans presented in Figure 19b and Figure 20b shows that the amplitude of the A_0_ mode wave obtained after filtering was 24 times or 27.6 dB lower than the amplitude of the total ultrasonic signal received by the air-coupled ultrasonic transducer.

Measurement of the phase velocity according to the above-described method obtained an evanescent wave velocity of 103.9 ± 0.7 m/s, which corresponds to the propagation velocity of the A_0_ mode in the PVC film. The obtained result confirms the efficiency of the described wave separation method based on 2D spatial–temporal filtering and illustrates the feasibility of air-coupled reception of the slow A_0_ Lamb wave mode.

**Figure 21:**
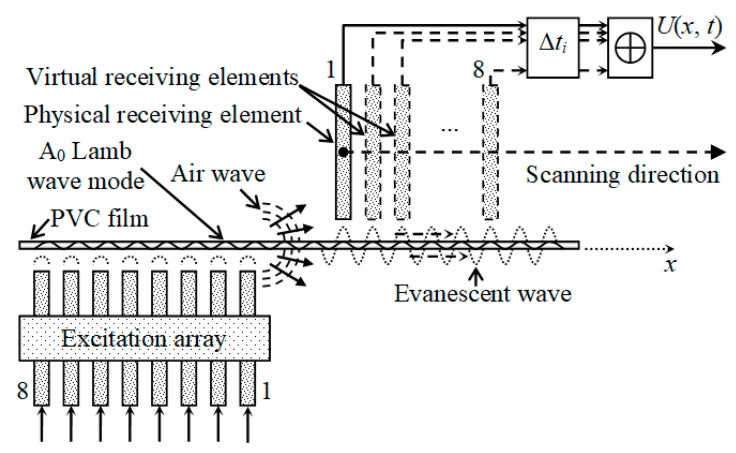
Reception of subsonic A_0_ mode by virtual receiving array.

To enhance the reception efficiency, a multi-element real or virtual array was used. The virtual array was obtained from the B-Scan data collected using the single receiving transducer (Figure 21).

**Figure 22:**
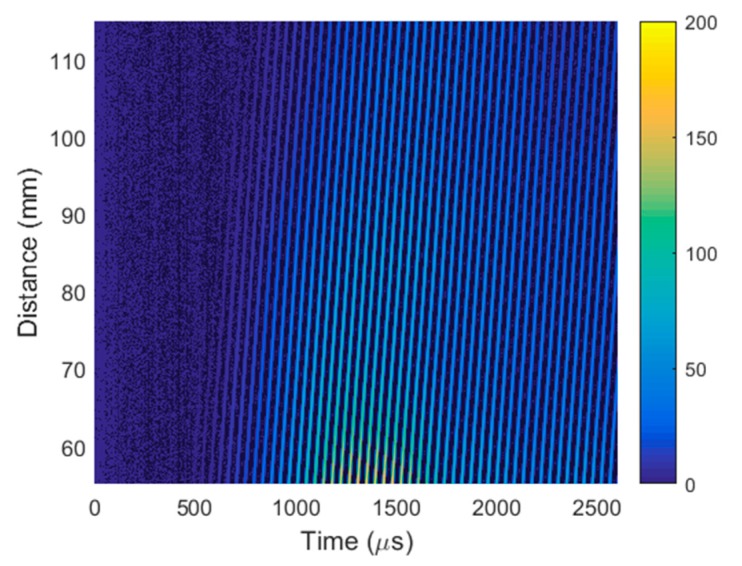
B-scan obtained using virtual air-coupled receiving phased array before filtering.

In this case, the B-scan signals were collected by one air-coupled transducer, and then the ultrasonic signals *u_i_*(*x_i_*, *t*) corresponding to the virtual array element center points *x_i_* were selected from the B-scan and summed in the time domain using the same delay times Δ*t_i_* as in the excitation array:

(9) $$U\left(x_{U},t\right)=\overset{8}{\underset{i=1}{\sum }}u_{i}\left(x_{i},t-\Delta t_{i}\right)$$

where the coordinate *x_U_* starts from the eighth array element center and indicates the position of the receiving array with respect to the excitation array (Figure 21). To measure the phase velocity of the propagating A_0_ mode, we need to obtain a new processed B-scan, i.e., a scan of the virtual array along the *x*-axis. For this purpose, after obtaining one signal *U*(*x_U_*, *t*) from the array, the coordinates of the virtual element centers *x_i_* are increased with a step of Δ*x* = 0.1 mm, i.e., the entire virtual array is moved and

(10) $$x_{j}=x_{i}+j\Delta x$$

where *j* is the number of scanning steps. In our case, the position of the virtual array *x_j_* changed from 55.1 mm to 115 mm. At each position, the delayed signals *u_i_* (*x_i_*, *t* *−* ⊗*t_i_*) were added according to the algorithm given by Equation (9), and the new processed B-scan *U*(*x_U_*, *t*) was obtained (Figure 22).

**Figure 23:**
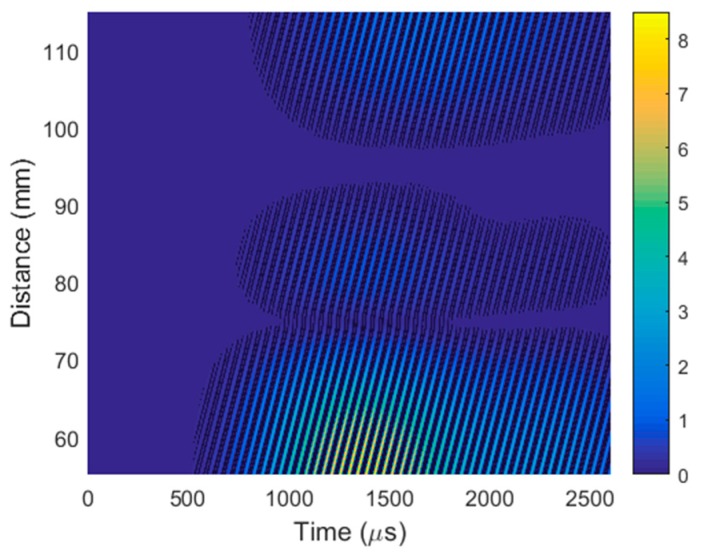
B-scan obtained by virtual air-coupled receiving phased array after 2D filtering.

For the efficient separation of the evanescent wave, the same 2D spatial–temporal filtering of the obtained B-scan was performed. The filtered B-Scan is shown in Figure 23.

**Figure 24:**
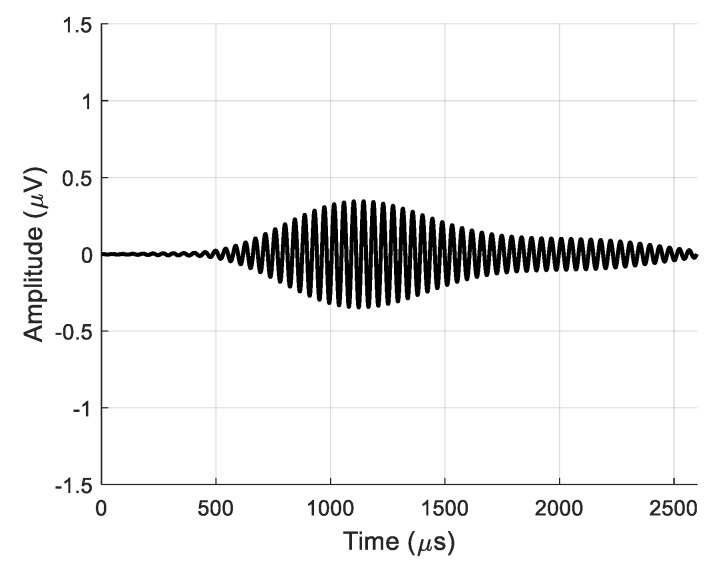
Signal from single air-coupled receiver obtained after 2D spatial–temporal filtering at *x* = 55.1 mm.

**Figure 25:**
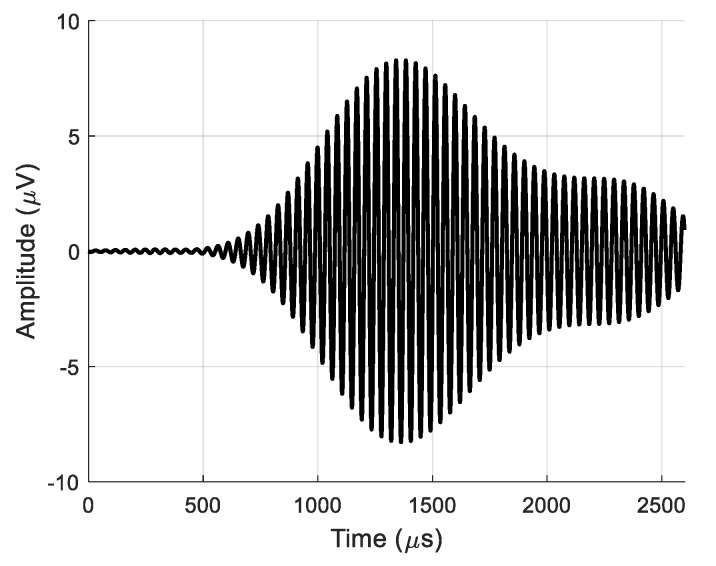
Signal from virtual air-coupled receiving array obtained after 2D spatial–temporal filtering at *x* = 55.1 mm.

For the estimation of the efficiency of the virtual receiving array, the real and virtual signals after 2D filtering at the same point *x* = 55.1 mm are displayed in Figure 24 and Figure 25, respectively. The maximum amplitude of the signal at the output of the single air-coupled receiver was *u* = 0.34·μV as shown in Figure 24. The signal at the output of the entire virtual array had an amplitude of *u* = 8.3·μV (24 times higher) as shown in Figure 25.

The phase velocity was determined from the filtered B-scan (Figure 23) according to Equation (4). The obtained phase velocity was 103.9 ± 0.7 m/s, which proves that the ultrasonic signal picked up by the air-coupled receiver was caused by the A_0_ mode propagating in the PVC film.

The proposed method based on the use of the evanescent wave and spatial–temporal filtering enables the reception of the subsonic A_0_ mode propagating in thin plates and films.

## 6. Discussion and Conclusions

The slow Lamb wave A_0_ mode can be excited in thin plates and films at frequencies lower than 50–100 kHz. Such waves do not radiate leaky waves into the surrounding air; therefore, there are no losses, and the waves can propagate over longer distances and be applied for non-destructive evaluation and material characterization. However, it is impossible to excite and receive such waves by air-coupled transducers deflected with respect to a sample surface by an angle given by Snell’s law.

In this study, we demonstrated that it is feasible to excite and pick up a subsonic A_0_ mode in plastic films by using air-coupled ultrasonic arrays. Air-coupled reception is based on the evanescent wave in air that accompanies the propagating A_0_ mode in a film. The evanescent wave propagates in close vicinity to the film surface; therefore, for the reception of this wave, the receiving transducer must be placed at a distance shorter than the wavelength of the ultrasonic wave in air. The efficiency of the reception can be enhanced by using a virtual array or a multi-element array. A virtual array was formed from the data collected during a B-scan obtained by a single air-coupled receiver. The signals measured at the points corresponding to the positions of the phase-matched array were recorded and processed. The processing started with the addition of the signals from all virtual array elements with proper delays. The transmitting array excited not only the A_0_ mode in the film, but also a direct wave in the air. This wave propagated with the velocity in air and was faster than the evanescent wave, which propagated with the velocity of the subsonic A_0_ mode. Therefore, the ultrasonic receiver picked up a mixture of two waves—the evanescent wave and the direct air wave. The direct wave is usually stronger than the evanescent wave; therefore, the additional signal-processing procedure based on the application of the 2D Fourier transform in the spatial–temporal domain was applied to efficiently separate the slower evanescent wave.

The obtained results can be useful for the development of novel air-coupled ultrasonic non-destructive testing and measurement techniques based on the application of subsonic guided waves.

## Figures and Tables

**Table 1:** Parameters of clear PVC film and Corning^®^ Gorilla^®^ Glass 6.

Parameter	Clear PVC film	Gorilla^®^ Glass 6
Density	*ρ* = 1400 kg/m^3^	*ρ* = 2400 kg/m^3^
Young’s modulus	*E* = 2156 MPa	*E* = 77.0 GPa
Poisson’s ratio	*ν* = 0.42	*ν* = 0.21
